# The role of education on Cancer amenable mortality among non-Hispanic blacks & non-Hispanic whites in the United States (1989–2018)

**DOI:** 10.1186/s12885-021-08633-7

**Published:** 2021-09-07

**Authors:** Alberto Barcelo, Linda Duffett-Leger, Maria Pastor-Valero, Juliana Pereira, Fernando A. B. Colugnati, Edward Trapido

**Affiliations:** 1 Universidade Federal de Juiz de Fora, Juiz de Fora, Minas Gerais, Brazil; 2 Department of Public Health Science, Miller School of Medicine, University of Miami, P.O. Box 414037, Miami Beach, FL 33141 USA; 3 Faculty of Nursing, University of Calgary, Calgary, Canada; 4 Departamento de Salud Pública, História de la Ciencia y Ginecología, Facultad de Medicina, Universidad Miguel Hernández de Elche, Elche, Spain; 5 Centro de Investigación Biomédica en Red de Epidemiología y Salud Publica (Ciberesp), Madrid, Spain; 6 Stanley S. Scott Cancer Center, LSU School of Public Health, New Orleans, USA

## Abstract

**Background:**

Cancer mortality in the U.S. has fallen in recent decades; however, individuals with lower levels of education experienced a smaller decline than more highly educated individuals. This analysis aimed to measure the influence of education lower than a high school diploma, on cancer amenable mortality among Non-Hispanic Whites (NHW) and Non-Hispanic Blacks (NHB) in the U.S. from 1989 to 2018.

**Methods:**

We analyzed data from 8.2 million death certificates of men and women who died from cancer between 1989 and 2018. We examined 5-year and calendar period intervals, as well as annual percent changes (APC). APC was adjusted for each combination of sex, educational level, and race categories (8 models) to separate the general trend from the effects of age.

**Results:**

Our study demonstrated an increasing mortality gap between the least and the most educated NHW and NHB males and females who died from all cancers combined and for most other cancer types included in this study. The gap between the least and the most educated was broader among NHW males and females than among NHB males and females, respectively, for most malignancies.

**Conclusions:**

In summary, we reported an increasing gap in the age-adjusted cancer mortality among the most and the least educated NHW and NHB between 25 and 74 years of age. We demonstrated that although NHB exhibited the greatest age-adjusted mortality rates for most cancer locations, the gap between the most and the least educated was shown for NHW.

**Supplementary Information:**

The online version contains supplementary material available at 10.1186/s12885-021-08633-7.

## Introduction

Low education is a strong predictor of high mortality in the United States (U.S.). The mortality gap between poorly and highly educated U.S. citizens has dramatically increased in recent years, particularly in specific subpopulations [1–3]. Mortality in the U.S. has declined more dramatically among the better educated than among less educated individuals [1–4].

Globally, cancer mortality increased from 7.7 million deaths in 2011 to 9.6 million deaths in 2017 [5, 6]. In the U.S., cancer mortality rates declined by 29%, from 1991 to 2017 [7]. However, it did not reduce equally in all U.S. population segments [8]. A greater prevalence of cancer and lower screening rates has been reported among Non-Hispanic Blacks (NHB) and individuals with less than high school education, compared to Non-Hispanic Whites (NHW) and their better-educated peers, respectively [9–17].

NHW individuals born in 1980s with a lower than grade 12 education, presented cancer amenable mortality rates almost three times higher than their counterparts born in 1955 with a comparable level of education; however, during the same period, no significant differences were found among the most educated NHW individuals [8]. A greater prevalence of cancer and lower screening rates have been found among NHB and those with education lower than high school compared to NHW and their highly educated peers, respectively [9–11]. Potentially modifiable social determinants of health, such as access to health care services and socioeconomic status (SES), are associated with cancer mortality, screening, and treatment disparities among vulnerable populations in the U.S. [8, 12–17].

Compared to neighborhoods with favorable economic indicators, higher breast cancer mortality rates have been reported in places where people with unfavorable social determinants of health clustered (lower education, lower household income, and higher unemployment and proportion of uninsured population) [18–21]. The difference in breast cancer mortality between NHW and NHB women has been linked to education, family income, access to primary care physicians, and mammography in communities [22]. Complex interactions between race and different SES such as education and neighborhood SES (nSES) were identified as a factor in higher breast cancer mortality among various racial groups [23].

Implementation of the Affordable Care Act (ACA) has had a measurable impact on improving early diagnosis of cancer among low income populations [24, 25], particularly in some of the more preventable or treatable cancers such as cervical, breast, and colorectal cancer [26]. The aim of this analysis was to measure the influence of education lower than a high school diploma on cancer amenable mortality among NHW and NHB in the U.S. between 1989 and 2018.

## Methods

### Study design and population

We used a time-series design to analyze data from death certificates of 8.2 million men and women who died from cancer between 1989 and 2018. We used for our analysis, death certificate data from the U.S. National Center for Health Statistics (NCHS) and U.S. population estimates by age, gender, and ethnicity from the U.S. Census Bureau [27]. Information on race and education for a death certificate is reported by funeral directors, family members or taken from medical records (1989 to 2018) [28].

### Variables and data collection

We selected mortality data due to specific cancer types, potentially amenable to appropriate health care, based on a modified version of the list published by Nolte et al. [29], as shown in Table 1.

**Table 1:** Causes of death considered amenable to health care

Cause of death	Age	International Classification of Disease		
		9th revision	10th revision	Comment
Malignant neoplasm of colon and rectum	0–74	153–4	C18–21	–
Malignant melanoma and other neoplasm of skin	0–74	173	C43-C44	–
Malignant neoplasm of the breast	0–74	174	C50	Females only
Malignant of the cervix uteri	0–74	180	C53	Females only
Malignant of the body of uterus	0–74	179–182	C54-C55	Females only
Malignant neoplasm of the testis	0–74	186	C62	Males only
Malignant neoplasm of the prostate	0–74	185	C61	Males only
Malignant neoplasm of the trachea, bronchus, and lung	0–74	162	C33-C34	–
Hodgkin’s disease	0–74	201	C81	–
Leukemia	0–74	204–8	C91-C05	–

Data on attained education and ethnicity were extracted from the death certificate databases. Counts of deaths due to colon, lung, breast, the body of the uterus, cervical, testis, prostate, Hodgkin’s disease, leukemia as well other cancers, were used to calculate age-adjusted mortality rates from 1989 to 2018, by year, sex, attained education (less than grade 12 or grade 12 and more) among NHW and NHB. We dichotomized the education variable into two groups of less than 12 years of education and 12 years or more of schooling, consistent with previous researchers [30, 31]. This approach was taken since the highest mortality rates were reported among those with less than 12 years of schooling [32, 33], suggesting that not completing a high school education was a critical threshold for the risk of mortality in the U.S. Furthermore, attaining a high school diploma is an important step in decreasing yearly mortality rates among adults in the U.S. [34]. Dichotomizing the education variable facilitated a simple presentation of data [30–33, 35].

Individuals with less than 25 or more than 74 years of age and those with missing or unknown education, or races other than NHW or NHB, were excluded.

Overall, only 5.9% of available records of cancer deaths among NHW and NHB were excluded from the analysis because the level of education was classified as unknown. From the 16.7 million overall cancer deaths in the U.S. (1989–2018), only 0.9%, the variable race was classified as unknown. We included 8.2 million deaths from a pool of 8.7 death certificates for NHW and NHB, who died from cancer between 1989 and 2018.

### Statistical analysis

Age-and-sex standardized rates were calculated using the 2000 U.S. census population as a standard (https://seer.cancer.gov/stdpopulations/stdpop.19ages.html). All analyses were restricted to individuals aged 25 to 74 years who died from cancers. We added deaths from prostate cancer to Nolte’s list since this was the only major cause of cancer absent from their list [36].

We examined 5-year and calendar period intervals and annual percent changes (APC) adjusted for each combination of sex, educational level, and race categories to separate the general trend from the effects of age. Data analysis was conducted using the Joinpoint Regression Program (https://surveillance.cancer.gov/joinpoint/) as well as SPSS-26 and Stata-14 [37–39].

## Results

**Fig. 1:**
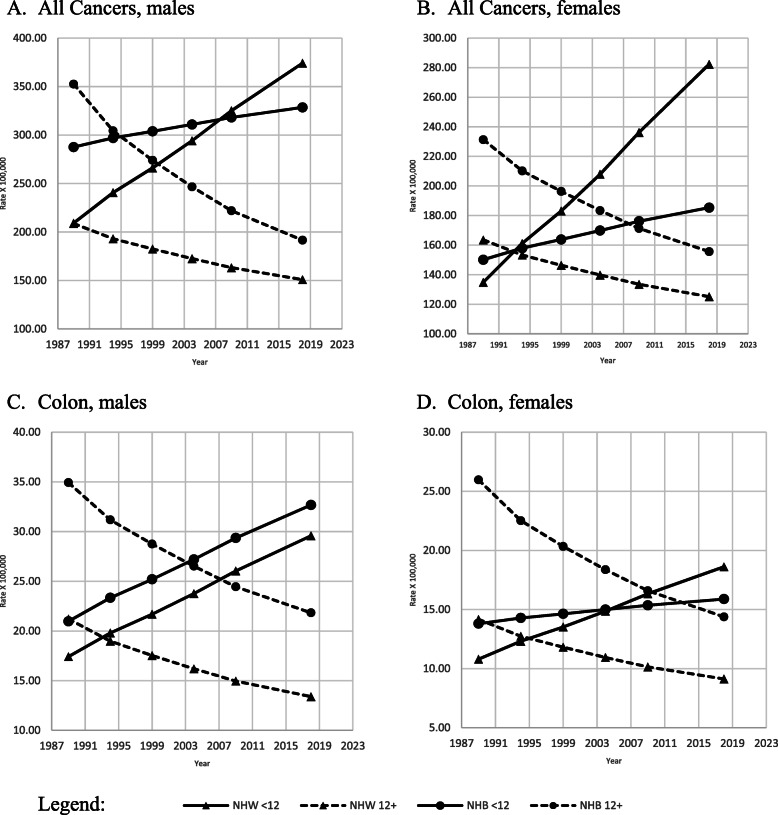
Adjusted amenable mortality rate (X100,000) due all cancer and cancer of colon and rectum, with Joinpoint Regression (modeled line of trends), among NHW & NHB by gender, period and education level. United States, 1989–2018. < 12: less than 12 years of education; 12+: 12 years of education or more. NHW: Non-Hispanic Whites; NHB: Non-Hispanic Blacks

All amenable age-adjusted cancer mortality rates, between 1989 and 2018, increased in both sexes and races with education less than 12 years, while they decreased among those with 12 or more years of education (Fig. 1 A-B).

Age-adjusted amenable colon and rectum cancer mortality rates were lower among the most educated than among their least educated peers for both races and sexes (Fig. 1, C-D). Age-adjusted mortality rates for colon and rectum cancer for both sexes and races increased among the least educated while decreasing among the most educated. These trends were statistically significant (Table 2).

**Table 2:** Average annual percentage change APC (and 95%-CI) in mortality by type of cancer among NHW and NHB, by gender and education level. United States, 1989–2018

				Level of education							
			< 12 Years					12 + Years			
	Race	APC	95%-CI				APC	95%-CI			
			Lower	Upper	t	p		Lower	Upper	t	p
		Males									
All cancers	NHW	2.0	1.0	3.1	5.6	0.0	−1.1	− 1.7	− 0.5	−5.2	0.0
	NHB	0.5	−0.9	1.9	0.9	0.4	−2.1	− 3.0	− 1.2	− 6.5	0.0
Colon & rectum	NHW	1.8	1.0	2.7	6.1	0.0	−1.6	−2.2	−0.9	− 6.7	0.0
	NHB	1.5	−0.2	3.3	2.5	0.1	−1.6	−2.6	−0.6	−4.4	0.0
Skin	NHW	2.6	1.2	4.0	5.3	0.0	−0.4	−1.2	0.4	− 1.4	0.2
	NHB	−0.6	−1.5	0.4	−1.7	0.2	−2.4	−2.9	−1.9	−13.1	0.0
Trachea, bronchus & lung	NHW	1.5	0.3	2.8	3.6	0.0	−2.1	−3.1	−1.1	−5.7	0.0
	NHB	−0.2	−1.8	1.5	− 0.3	0.8	− 3.2	−4.4	−2.0	−7.2	0.0
Hodgkin’s disease	NHW	1.1	−1.0	3.2	1.4	0.2	−2.8	−3.3	−2.3	−16.9	0.0
	NHB	0.3	−1.7	2.3	0.4	0.7	−3.0	−4.1	−1.8	−7.1	0.0
Leukemia	NHW	1.8*	0.4	3.1	3.7	0.0	−1.0	−2.0	−0.1	−3.1	0.0
	NHB	0.7	−0.9	2.3	1.2	0.3	−1.6	−2.5	−0.7	−5.1	0.0
Testis	NHW	4.3	1.3	7.4	4.0	0.0	0.3	−0.5	1.0	1.0	0.4
	NHB	1.4	−3.4	6.4	0.8	0.5	0.1	−2.6	2.8	0.1	1.0
Prostate	NHW	−0.3	−0.4	−0.2	−7.3	0.0	−2.5	−3.3	−1.6	− 8.1	0.0
	NHB	−0.3	−2.2	1.6	−0.5	0.6	−2.7	−3.8	−1.6	−6.7	0.0
Other types	NHW	2.8	1.8	3.8	7.8	0.0	−0.2	−0.7	0.3	−1.2	0.3
	NHB	0.9	−0.2	2.0	2.4	0.1	−1.3	−2.0	−0.6	−5.1	0.0
		Females									
All cancers	NHW	2.6	1.3	3.9	5.4	0.0	−0.9	−1.6	−0.3	−3.9	0.0
	NHB	0.7	−0.2	1.7	2.1	0.1	−1.4	−2.1	−0.6	−5.2	0.0
Colon & rectum	NHW	1.9	0.9	2.9	5.4	0.0	−1.5	−2.2	−0.9	−6.4	0.0
	NHB	0.5	−0.6	1.6	1.2	0.3	−2.0	−2.9	−1.1	−6.0	0.0
Skin	NHW	2.6	1.0	4.3	4.5	0.0	−0.4	−1.3	0.4	− 1.4	0.2
	NHB	1.1	−0.3	2.5	2.1	0.1	−2.1	−3.0	−1.3	−6.9	0.0
Trachea, bronchus & lung	NHW	3.4	1.6	5.3	5.2	0.0	−0.9	−2.2	0.3	−2.1	0.1
	NHB	1.1	−0.6	2.8	1.8	0.2	−1.9	−3.1	−0.7	−4.3	0.0
Hodgkin’s Disease	NHW	2.2	0.9	3.6	4.6	0.0	−2.7	−4.4	−1.0	−4.5	0.0
	NHB	0.8	−1.0	2.7	1.2	0.3	−2.5	−3.8	−1.3	−5.5	0.0
Leukemia	NHW	1.8	0.5	3.1	3.8	0.0	−1.1	−1.9	−0.2	−3.4	0.0
	NHB	0.3	−0.6	1.3	1.0	0.4	−1.4	−2.0	−0.7	−5.9	0.0
Breast	NHW	1.0	−0.3	2.2	2.2	0.1	−1.8	−2.2	−1.3	− 11.3	0.0
	NHB	0.5	−0.5	1.4	1.4	0.2	−1.4	−2.1	−0.7	−5.3	0.0
Cervix uteri	NHW	3.1	2.0	4.2	7.9	0.0	−0.4	−0.9	0.2	−1.9	0.1
	NHB	−0.4	−1.3	0.4	−1.4	0.2	−2.2	−3.0	−1.4	−7.4	0.0
Body of uterus	NHW	3.1	2.3	4.0	10.3	0.0	1.3	1.0	1.5	15.9	0.0
	NHB	2.3	1.8	2.7	14.7	0.0	1.5	1.1	1.9	10.1	0.0
Other types	NHW	2.4	1.2	3.7	5.7	0.0	−0.5	−1.2	0.1	−2.3	0.1
	NHB	0.7	−0.1	1.6	2.4	0.1	−1.1	−1.8	−0.4	−4.5	0.0

*< 12* less than 12 years of education; *12+* 12 years of education or more

*NHW* Non-Hispanic Whites; *NHB* Non-Hispanic Blacks

*APC* Average annual percentage change

*95%-CI* 95% Confidence intervals

**Fig. 2:**
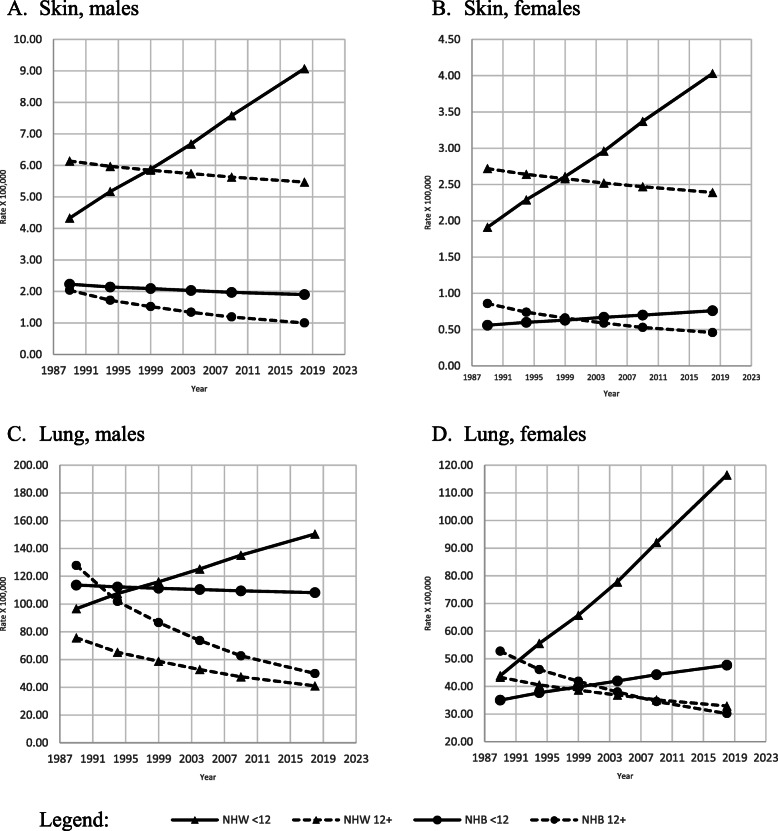
Adjusted amenable mortality rate (X100,000) due to cancer of the skin and cancer of the trachea, bronchus, and lung with Joinpoint Regression (modeled line of trends) among NHW & NHB by gender, period and education level. United States, 1989–2018. < 12: less than 12 years of education; 12+: 12 years of education or more. NHW: Non-Hispanic Whites; NHB: Non-Hispanic Blacks

Age-adjusted skin cancer mortality rates (Fig. 2, A-B) were two to three times higher among NHW than their NHB peers. NHW men and women with lower levels of education presented lower mortality rates than their higher-educated peers between 1989 and 1998; however, trends for both NHW genders reversed in 1999–2018, showing consistently higher age-adjusted mortality rates among the less educated compared to the most educated.

Age-adjusted mortality due to lung and trachea cancer was three times higher among the less educated compared their most educated peers (Fig. 2, C-D). The APC analysis indicated statistically significant decreasing trends for NHW and NHB males and NHB women with 12 or more years of education (Table 2).

**Fig. 3:**
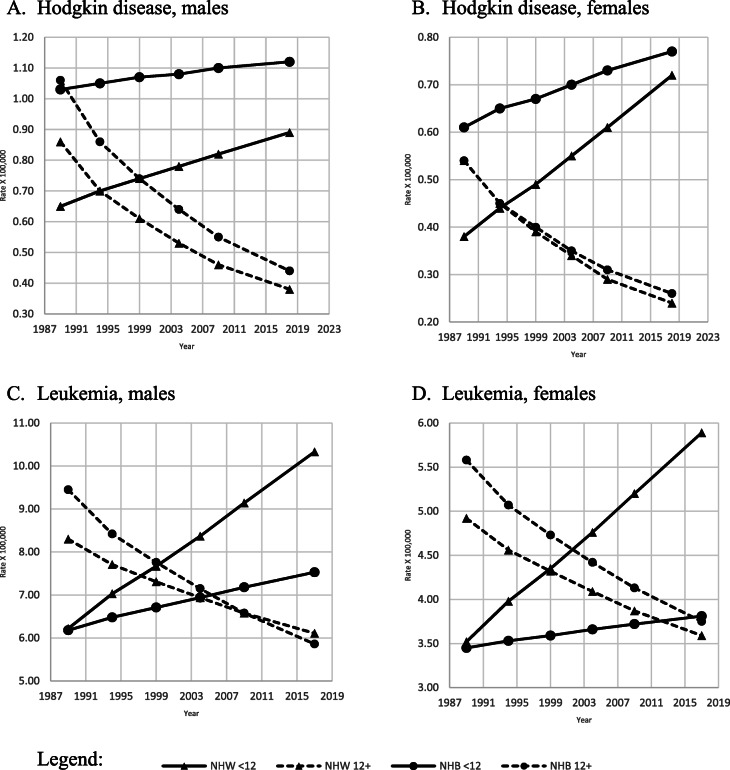
Adjusted amenable mortality rate (X100,000) due to Hodgkin’s disease and leukemia, with Joinpoint Regression (modeled line of trends), among NHW & NHB by gender, period and education level. United States, 1989–2018. < 12: less than 12 years of education; 12+: 12 years of education or more. NHW: Non-Hispanic Whites; NHB: Non-Hispanic Blacks

Age-adjusted amenable mortality rates for Hodgkin’s disease and leukemia were the lowest for NHW males and females among the most educated, and they were comparably low for NHB of the same gender and education (Fig. 3, A-B, C-D). The least educated NHB men and women presented the highest age-adjusted mortality rates for Hodgkin’s disease during the study period.

**Fig. 4:**
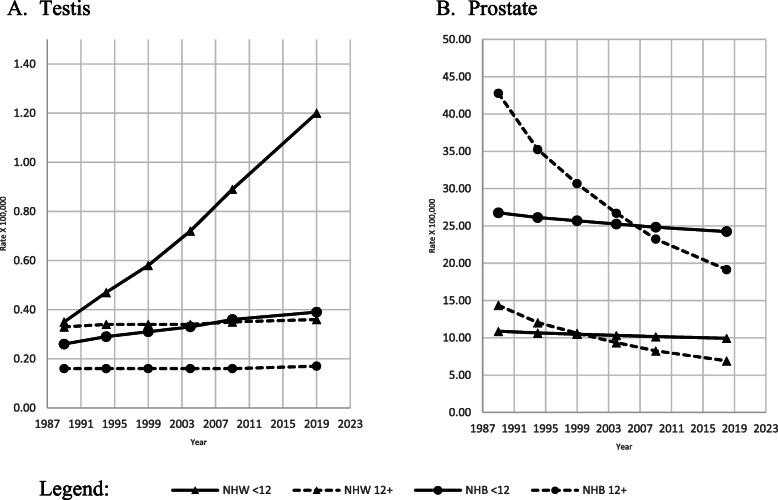
Adjusted amenable mortality rate (X100,000) due to cancer of the testis and prostate cancer with Joinpoint Regression (modeled line of trends), among NHW & NHB males by period and education level. United States, 1989–2018. < 12: less than 12 years of education; 12+: 12 years of education or more. NHW: Non-Hispanic Whites; NHB: Non-Hispanic Blacks

Age-adjusted cancer of the testis mortality rates (Fig. 4, A) was the highest among the less educated NHW and NHB than the most educated peers. NHB men had age-adjusted cancer of the testis rates between 50% and 66% lower than NHW males. The age-adjusted mortality rate for prostate cancer (Fig. 4, B) was more than double among NHB men than NHW men.

**Fig. 5:**
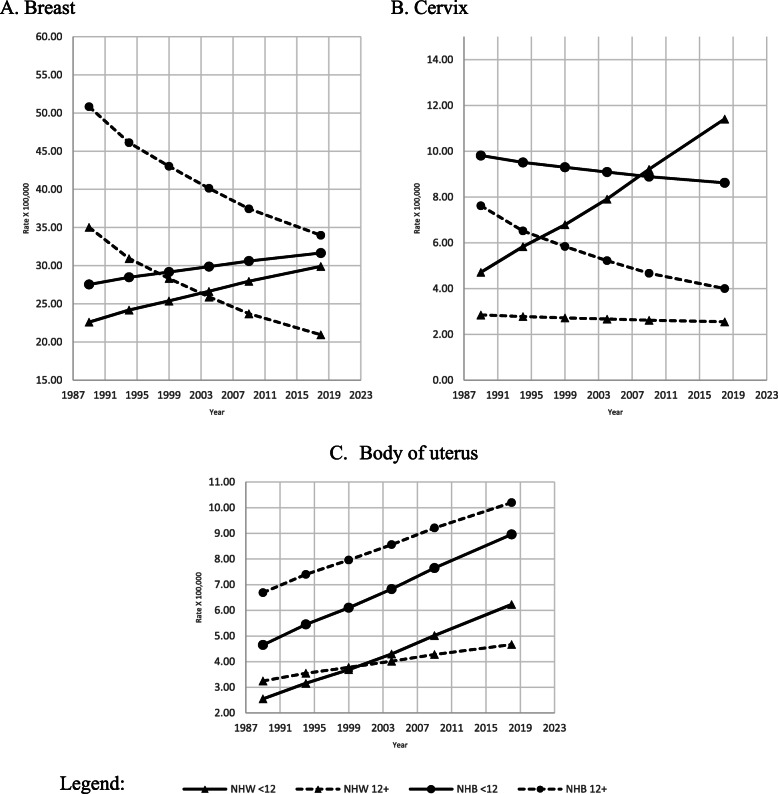
Adjusted amenable mortality rate (X100,000) due to breast, cervix uteri, and body of uterus cancer, with Joinpoint Regression (modeled line of trends), among NHW & NHB females by period and education level. United States, 1989–2018. < 12: less than 12 years of education; 12+: 12 years of education or more. NHW: Non-Hispanic Whites; NHB: Non-Hispanic Blacks

Breast cancer mortality rates (Fig. 5, A) were the lowest among the most educated NHW, beginning 2000 when there was an increasing trend for the least educated NHW and NHB, respectively. The highest mortality rates during the study period were observed among the most educated NHB; however, there was a significant decreasing trend (APC − 1.4, *p* < 0.01) among NHB women with an education of 12 years or higher (Table 2).

Age-adjusted mortality rates for cancer of the cervix (Fig. 5, B) were two to three times higher among the least educated than their most educated peers of both races, the gap was the biggest among NHW. The APC analysis indicated a statistically significant increasing trend among the least educated NHW (Table 2).

The age-adjusted mortality rate for the body of the uterus (Fig. 5 C) increased among females of both races and education levels; however, NHB with an education of 12 years and higher had higher mortality rates than all other groups. All four increasing trends were statistically significant (Table 2).

**Fig. 6:**
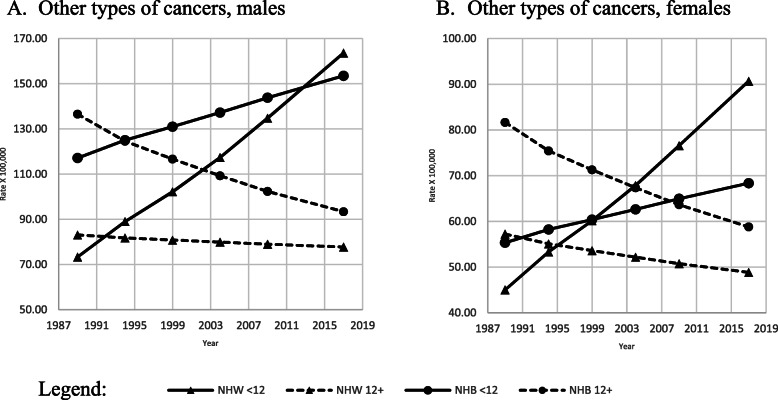
Adjusted amenable mortality rate (X100,000) due to all other types of cancer, with Joinpoint Regression (modeled line of trends), among NHW & NHB, by gender, period and education level. United States, 1989–2018. < 12: less than 12 years of education; 12+: 12 years of education or more. NHW: Non-Hispanic Whites; NHB: Non-Hispanic Blacks

All other cancer age-adjusted mortality rates increased (Fig. 6 A-B) among the less educated and decreased among the most educated of both races and genders. APC indicated a statistically significant increasing trend among the less educated NHW men and women (Table 2).

## Discussion

Our study demonstrated an increased mortality gap between the least and the most educated who died from most types of cancers. The gap between the least and most educated was broader for most malignancies among NHW than among NHB, respectively, suggesting delayed death from some of the most frequent cancers among individuals in the U.S. with grade 12 or higher education [40].

Among the limitation of our study was the lack of additional variables, such as income or access to health insurance. Moreover, we relayed on the level of education reported on death certificates, which are subject to reporter’s recall bias. Besides that, there are controversial reports regarding the reliability of the level of education information provided by death certificates. Research has demonstrated that a proportion of cases who attended high school are reported as graduated, even though they did not achieve a diploma. That would overestimate the number of high school graduates in our sample, especially among NHB. But this bias, however, is unlikely to change our results [41, 42] since the difference we found appears to be consistent across time, race, and cancer type. Analyzing age-and-sex adjusted 5-year periods eliminated slight variations that might have occurred in a particular year, state, or race. While we cannot rule out a birth cohort influence, on many cancer type mortality rates, since recent research has demonstrated an increasing trend of cancer incidence among young adults in the US due to variation in risk factor frequency [43] the impact on our results are likely negligible.

The strength of using the level of education as an SES factor in health research lies in its lifetime stability and inclusiveness of measurement. Education is less likely to be influenced by adulthood health or income. Higher education is considered a good predictor of improved lifestyles and behaviors, better jobs, housing, working conditions, and income [4, 44–46]. Conversely, the effect of education might have different meanings and returns across the racial spectrum of the U.S. population. Having less than a 12th grade education is one of the greatest SES contributors to premature mortality when considering poverty, low social support, income, racial segregation, and health insurance [11, 30, 31, 47].

Implementing evidence-based programs focused on social factors such as education and environmental context to reduce cancer screening disparities between NHB and NHW is recommended [40, 48]. Although recent statistics suggest lower (or comparable) death rates among NHW and NHB due to certain cancers such as breast, prostate, cervix, colon and rectum [7], our data showed that this decrease was only apparent in some segments of the U.S. population, i.e., the most educated NHW. In particular, melanoma of the skin and cancer of the testis has been reported to be more frequent among NHW than among NHB [49].

Many risk factors have been identified as causing cancer mortality disparities among U.S. racial groups [50]. Lower SES was related to lower awareness for breast cancer symptoms and prevention [17, 51], resulting in more advanced stage cancer presentations at diagnosis than disparities in treatment or access to health insurance [16, 17, 22, 52–55].

Age-adjusted cancer mortality of three types of cancers (leukemia, breast, and body of the uterus) was found to be higher among the most educated NHB than their least educated peers. Age-adjusted mortality rates for the body of the uterus cancer showed a steady increase in all subpopulation groups. Although our data suggest the influence of education (attaining 12 years or more) on mortality rates for most major cancers, this factor did not appear to have the same detrimental effect on cancer deaths from leukemia, breast, and body of the uterus among NHB women. While access to oral contraceptive use [56] or hormone replacement therapy [57] may play a role in this issue, it is likely an interaction of other important risk factors. Incidence studies have demonstrated lower breast cancer incidence among NHB women than NHW women [7, 58]. Additionally, breast cancer mortality rates have been reported to be more than 30% higher among NHB than among NHW women [47]. It is also possible that we missed some of the known race specific breast cancer differences since we did not analyze the age-specific rate by race and education. For instance, the presentation of triple negative aggressive breast cancer among young age NHB women that appears later in life among NHW women [59, 60].

A lower proportion of women of color compared to NHW women reported having mammography during the previous 2 years [61]. Our data showed that breast cancer and cancer of the uterus age-adjusted mortality rates among the most educated NHB women, between the age of 25 and 44 years (data not shown), were slightly lower than their least educated peers. These findings indicate that the increase in breast and uterine cancer mortality among the most educated NHB may be the result of greater mortality among postmenopausal women (data not shown) compared to women before the age of 45 years of age, due to greater likelihood of hormone replacement therapy use among members of this group. A decreased use of hormone replacement therapy has been related to a lower incidence of breast cancer among NHW women [62]. Other factors that may be involved in the observed differences are limited availability of community mammography facilities [22], increased delays in cancer treatment among women of color, and lack of health insurance [63]. NHB women living in low SES areas showed poorer breast cancer outcomes than NHW women who were recipients of the same government-subsidized health services, even after adjusting by the level of education [64].

Research exploring the complex multi-factorial causes such as biological, social, health system factors, hormone replacement therapy, and contraceptive use may help explain our findings [49, 56, 57, 65–71].

The Global Burden of Disease (GDB) study showed that country cancer profiles depend on the combination of different exposures to risk factors and socioeconomic and health care access conditions [72]. The prevention of cancer mortality in underserved populations requires increasing healthcare coverage and outreach programs to overcome barriers related to stigma, clinician implicit bias, and nihilism [73]. Furthermore, this disparity demonstrates a need for culturally appropriate cancer prevention interventions tailored to the unique demands of the most vulnerable groups in the U.S. population for success [40].

In summary, we reported an increasing gap in the age-adjusted cancer mortality among the most and the least educated NHW and NHB between 25 and 74 years of age. These deaths are amenable to access to quality health care improvement. We also found that the most educated NHW had lower mortality rates for most malignancies than the less-educated NHW and their most educated and least educated NHB peers.

Although national statistics reported that the overall declines in cancer death rates in the U.S. were the result of significant decreases in four leading cancers (lung, colorectal, breast, and prostate cancers) [7], our data showed an increasing trend of cancer mortality among the least educated NHW and NHB for the same four (and others) types of cancers. These findings suggest notable educational and racial disparities in the prevention of cancer incidence, treatment, and mortality among some segments of the U.S. population, which can probably be circumvented by increasing access to prevention, screening, and enhanced quality treatment of cancer among some sub-populations. We also demonstrated that although NHB exhibited the highest age-adjusted mortality rates for most cancer locations, the greatest gap between the most and the least educated was shown for NHW.

Decreasing educational and racial health disparities may improve access to screening, as well as preventive and quality care for a large segment of the population and further reduce premature cancer mortality in the U.S.

## Supplementary Information

**Additional file 1: Table S1**. Adjusted amenable cancer mortality rates (X100,000) and 95% Confidence Intervals (CI) among NHW and NHB by gender and education level. United States, 1989–2018. **Table S2**. Adjusted amenable colon and rectum* mortality rates (X100,000) and 95% Confidence Intervals (CI) among NHW and NHB by gender and education level. United States, 1989–2018. **Table S3**. Adjusted amenable skin cancer* mortality rates (X100,000) and 95% Confidence Intervals (CI) among NHW and NHB by gender and education level. United States, 1989–2018. **Table S4**. Adjusted amenable lung and trachea cancer* mortality rates (X100,000) and 95% Confidence Intervals (CI) among NHW and NHB men by education level. United States, 1989–2018. **Table S5**. Adjusted amenable Hodgkin’s disease* mortality rates (X100,000) and 95% Confidence intervals (CI) among NHW and NHB by gender and education level. United States, 1989–2018. **Table S6**. Adjusted amenable leukemia* mortality rates (X100,000 and 95% Confidence intervals (CI) among NHW and NHB (25–74 years of age) by gender and education level. United States, 1989–2018. **Table S7**. Adjusted amenable testis cancer* rates (X100,000 and 95% Confidence intervals (CI) among NHW and NHB men by education level. United States, 1989–2018. **Table S8**. Adjusted amenable prostate cancer* mortality rates (X100,000 and 95% Confidence intervals (CI) among NHW and NHB men by education level. United States, 1989–2018. **Table S9**. Adjusted amenable breast cancer* mortality rates (X100,000) and 95% Confidence intervals (CI) among NHW and NHB by education level. United States, 1989–2018. **Table S10**. Adjusted amenable cervix cancer* mortality rates (X100,000) and 95% Confidence intervals (CI) among NHW and NHB by education level. United States, 1989–2018. **Table S11**. Adjusted amenable body of uterus cancer* mortality rates (X100,000) and 95% Confidence intervals (CI) among NHW and NHB female by education level. United States, 1989–2018. **Table S12**. Adjusted amenable other type cancers* mortality rates (X100,000) and 95% Confidence intervals (CI) among NHW and NHB by gender and education level. United States, 1989–2018.

## Data Availability

The datasets generated and analyzed during the current study are available in this repository https://drive.google.com/drive/folders/1HHjd0ZOQlAKKwFmsUYt-OSdAh2C485QZ?usp=sharing in SPSS format.

## Abbreviations

The U.S.: United States of America
NCD: Non-communicable diseases
NHW: Non-hispanic whites
NHB: Non-hispanic whites
L.E.: Life expectancy
ACP: Annual percentage changes
95%-CI: 95% Confidence intervals
SES: Socioeconomic status
nSES: Neighborhood SES
GBD: Global burden of disease
ACA: Affordable care act
