# Supplementary material for: The role of education on Cancer amenable mortality among non-Hispanic blacks & non-Hispanic whites in the United States (1989–2018)
Source: BMC Cancer. 2021 Sep 7;21:907. doi: 10.1186/s12885-021-08633-7 (PMC8425171; doi:10.1186/s12885-021-08633-7)
Supplement: Supplementary file 1 — Additional file 1: Table S1. Adjusted amenable cancer mortality rates (X100,000) and 95% Confidence Intervals (CI) among NHW and NHB by gender and education level. United States, 1989–2018. Table S2. Adjusted amenable colon and rectum* mortality rates (X100,000) and 95% Confidence Intervals (CI) among NHW and NHB by gender and education level. United States, 1989–2018. Table S3. Adjusted amenable skin cancer* mortality rates (X100,000) and 95% Confidence Intervals (CI) among NHW and NHB by gender and education level. United States, 1989–2018. Table S4. Adjusted amenable lung and trachea cancer* mortality rates (X100,000) and 95% Confidence Intervals (CI) among NHW and NHB men by education level. United States, 1989–2018. Table S5. Adjusted amenable Hodgkin’s disease* mortality rates (X100,000) and 95% Confidence intervals (CI) among NHW and NHB by gender and education level. United States, 1989–2018. Table S6. Adjusted amenable leukemia* mortality rates (X100,000 and 95% Confidence intervals (CI) among NHW and NHB (25–74 years of age) by gender and education level. United States, 1989–2018. Table S7. Adjusted amenable testis cancer* rates (X100,000 and 95% Confidence intervals (CI) among NHW and NHB men by education level. United States, 1989–2018. Table S8. Adjusted amenable prostate cancer* mortality rates (X100,000 and 95% Confidence intervals (CI) among NHW and NHB men by education level. United States, 1989–2018. Table S9. Adjusted amenable breast cancer* mortality rates (X100,000) and 95% Confidence intervals (CI) among NHW and NHB by education level. United States, 1989–2018. Table S10. Adjusted amenable cervix cancer* mortality rates (X100,000) and 95% Confidence intervals (CI) among NHW and NHB by education level. United States, 1989–2018. Table S11. Adjusted amenable body of uterus cancer* mortality rates (X100,000) and 95% Confidence intervals (CI) among NHW and NHB female by education level. United States, 1989–2018. Table S12. Adjusted amenable other ty [file 12885_2021_8633_MOESM1_ESM.docx]

# Supplemental data

Table1. Adjusted amenable cancer mortality rates (X100,000) and 95% Confidence Intervals (CI) among NHW and NHB by gender and education level. United States, 1989-2018.

|  | Education level | | | | | | | | | | | |
| --- | --- | --- | --- | --- | --- | --- | --- | --- | --- | --- | --- | --- |
|  | <12 years | | | | ≥12 years | | | | | | | |
|  | Rate | | 95%-CI | | Rate | | | | 95%-CI | | | |
| Period | Crude | Adjusted | Low | High | Crude | | Adjusted | | Low | | High | |
|  | NHW, males | | | | | | | | | | | |
| 1989-1993 | 310.54 | 202.45 | 200.18 | 204.74 | 165.05 | | 194.12 | | 192.79 | | 195.47 | |
| 1994-1998 | 331.36 | 228.91 | 226.23 | 231.63 | 173.77 | | 199.97 | | 198.69 | | 201.25 | |
| 1999-2003 | 475.02 | 293.86 | 290.02 | 297.73 | 183.97 | | 193.11 | | 191.89 | | 194.34 | |
| 2004-2008 | 469.63 | 314.55 | 310.31 | 318.83 | 182.65 | | 174.14 | | 173.04 | | 175.24 | |
| 2009-2013 | 498.99 | 335.80 | 331.09 | 340.57 | 193.51 | | 162.48 | | 161.50 | | 163.46 | |
| 2014-2018 | 516.37 | 335.11 | 330.10 | 340.18 | 198.11 | | 146.63 | | 145.77 | | 147.51 | |
|  | NHB, males | | | | | | | | | | | |
| 1989-1993 | 367.79 | 250.40 | 244.69 | 256.22 | 157.63 | | 307.54 | | 300.44 | | 314.76 | |
| 1994-1998 | 443.80 | 331.61 | 324.39 | 338.97 | 182.83 | | 336.47 | | 329.77 | | 343.27 | |
| 1999-2003 | 462.10 | 328.54 | 320.67 | 336.56 | 182.51 | | 282.63 | | 277.34 | | 287.98 | |
| 2004-2008 | 421.42 | 310.32 | 302.61 | 318.18 | 179.77 | | 250.56 | | 246.02 | | 255.16 | |
| 2009-2013 | 434.08 | 327.38 | 319.12 | 335.82 | 191.91 | | 220.08 | | 216.53 | | 223.67 | |
| 2014-2018 | 416.92 | 295.96 | 288.02 | 304.08 | 185.47 | | 187.31 | | 184.49 | | 190.17 | |
|  | NHW, females | | | | | | | | | | |  |
| 1989-1993 | 212.95 | 134.20 | 132.24 | 136.19 | 142.62 | 150.88 | | 149.81 | | 151.97 | |  |
| 1994-1998 | 226.48 | 144.62 | 142.48 | 146.78 | 153.66 | 160.23 | | 159.16 | | 161.30 | |  |
| 1999-2003 | 353.51 | 209.19 | 205.69 | 212.74 | 159.36 | 154.30 | | 153.28 | | 155.32 | |  |
| 2004-2008 | 370.18 | 224.29 | 220.40 | 228.24 | 156.57 | 141.75 | | 140.82 | | 142.69 | |  |
| 2009-2013 | 387.74 | 241.18 | 236.73 | 245.70 | 162.62 | 132.78 | | 131.93 | | 133.64 | |  |
| 2014-2018 | 406.32 | 253.51 | 248.47 | 258.65 | 165.22 | 120.86 | | 120.09 | | 121.64 | |  |
|  | NHB, females | | | | | | | | | | |  |
| 1989-1993 | 193.88 | 136.93 | 132.77 | 141.19 | 126.46 | 207.55 | | 202.74 | | 212.44 | |  |
| 1994-1998 | 237.04 | 169.65 | 164.63 | 174.80 | 147.73 | 228.72 | | 224.31 | | 233.19 | |  |
| 1999-2003 | 236.73 | 168.78 | 163.52 | 174.18 | 151.37 | 202.41 | | 198.79 | | 206.07 | |  |
| 2004-2008 | 235.11 | 174.71 | 169.05 | 180.52 | 148.85 | 181.66 | | 178.56 | | 184.81 | |  |
| 2009-2013 | 245.16 | 183.04 | 177.03 | 189.22 | 158.77 | 168.85 | | 166.22 | | 171.51 | |  |
| 2014-2018 | 242.88 | 168.54 | 162.55 | 174.70 | 159.65 | 154.58 | | 152.32 | | 156.86 | |  |

Table2. Adjusted amenable colon and rectum* mortality rates (X100,000) and 95% Confidence Intervals (CI) among NHW and NHB by gender and education level. United States, 1989-2018.

|  | Education level | | | | | | | | | | | |
| --- | --- | --- | --- | --- | --- | --- | --- | --- | --- | --- | --- | --- |
|  | < 12 Years | | | | ≥ 12 Years | | | | | | | |
|  | Rate | | 95%-CI | | Rate | | | | 95%-CI | | | |
| Period | Crude | Adjusted | Low | High | Crude | | Adjusted | | Low | | High | |
|  | NHW, males | | | | | | | | | | | |
| 1989-1993 | 26.80 | 17.06 | 16.41 | 17.72 | 17.02 | | 20.12 | | 19.69 | | 20.55 | |
| 1994-1998 | 27.98 | 18.93 | 18.17 | 19.71 | 17.26 | | 19.93 | | 19.53 | | 20.34 | |
| 1999-2003 | 39.37 | 23.67 | 22.61 | 24.78 | 17.78 | | 18.63 | | 18.25 | | 19.02 | |
| 2004-2008 | 37.37 | 24.89 | 23.71 | 26.12 | 16.36 | | 15.55 | | 15.23 | | 15.88 | |
| 2009-2013 | 39.33 | 26.77 | 25.43 | 28.16 | 16.99 | | 14.41 | | 14.12 | | 14.71 | |
| 2014-2018 | 40.24 | 27.02 | 25.57 | 28.53 | 17.51 | | 13.57 | | 13.30 | | 13.85 | |
|  | NHB, males | | | | | | | | | | | |
| 1989-1993 | 26.30 | 17.32 | 15.87 | 18.88 | 14.84 | | 29.44 | | 27.25 | | 31.74 | |
| 1994-1998 | 35.97 | 26.56 | 24.56 | 28.69 | 18.53 | | 34.15 | | 32.03 | | 36.35 | |
| 1999-2003 | 38.94 | 27.28 | 25.07 | 29.66 | 19.97 | | 30.56 | | 28.85 | | 32.34 | |
| 2004-2008 | 37.64 | 27.68 | 25.41 | 30.12 | 19.75 | | 27.25 | | 25.77 | | 28.78 | |
| 2009-2013 | 40.17 | 30.97 | 28.42 | 33.71 | 21.29 | | 23.89 | | 22.74 | | 25.07 | |
| 2014-2018 | 39.15 | 28.53 | 26.05 | 31.19 | 21.27 | | 21.36 | | 20.42 | | 22.33 | |
|  | NHW, females | | | | | | | | | | |  |
| 1989-1993 | 19.18 | 11.09 | 10.56 | 11.65 | 12.75 | 13.41 | | 13.09 | | 13.73 | |  |
| 1994-1998 | 18.89 | 11.15 | 10.59 | 11.74 | 13.04 | 13.56 | | 13.25 | | 13.87 | |  |
| 1999-2003 | 26.82 | 14.93 | 14.03 | 15.88 | 12.76 | 12.37 | | 12.08 | | 12.66 | |  |
| 2004-2008 | 25.92 | 15.30 | 14.31 | 16.35 | 11.61 | 12.37 | | 12.08 | | 12.66 | |  |
| 2009-2013 | 25.91 | 16.19 | 15.05 | 17.41 | 11.61 | 10.56 | | 10.30 | | 10.81 | |  |
| 2014-2018 | 28.31 | 18.15 | 16.78 | 19.60 | 12.22 | 9.26 | | 9.05 | | 9.49 | |  |
|  | NHB, females | | | | | | | | | | |  |
| 1989-1993 | 12.53 | 11.39 | 13.77 | 13.06 | 22.87 | 21.25 | | 24.57 | | 12.53 | |  |
| 1994-1998 | 15.57 | 14.14 | 17.12 | 14.97 | 24.37 | 22.92 | | 25.88 | | 15.57 | |  |
| 1999-2003 | 15.20 | 13.70 | 16.82 | 15.92 | 21.91 | 20.71 | | 23.15 | | 15.20 | |  |
| 2004-2008 | 15.34 | 13.74 | 17.09 | 14.57 | 18.06 | 17.08 | | 19.07 | | 15.34 | |  |
| 2009-2013 | 16.02 | 14.29 | 17.93 | 15.05 | 15.90 | 15.10 | | 16.73 | | 16.02 | |  |
| 2014-2018 | 14.16 | 12.47 | 16.04 | 14.85 | 14.39 | 13.71 | | 15.10 | | 14.16 | |  |

Table 3. Adjusted amenable skin cancer* mortality rates (X100,000) and 95% Confidence Intervals (CI) among NHW and NHB by gender and education level. United States, 1989-2018.

|  | Education level | | | | | | | | | | | |
| --- | --- | --- | --- | --- | --- | --- | --- | --- | --- | --- | --- | --- |
|  | < 12 Years | | | | ≥ 12 Years | | | | | | | |
|  | Rate | | 95%-CI | | Rate | | | | 95%-CI | | | |
| Period | Crude | Adjusted | Low | High | Crude | | Adjusted | | Low | | High | |
|  | NHW, males | | | | | | | | | | | |
| 1989-1993 | 5.50 | 4.24 | 3.88 | 4.63 | 5.04 | | 5.74 | | 5.51 | | 5.97 | |
| 1994-1998 | 5.76 | 4.63 | 4.22 | 5.07 | 5.39 | | 5.98 | | 5.76 | | 6.20 | |
| 1999-2003 | 9.12 | 6.66 | 6.04 | 7.33 | 5.91 | | 6.03 | | 5.82 | | 6.25 | |
| 2004-2008 | 9.64 | 7.21 | 6.54 | 7.94 | 6.34 | | 5.99 | | 5.79 | | 6.20 | |
| 2009-2013 | 10.68 | 7.96 | 7.20 | 8.80 | 6.93 | | 5.95 | | 5.76 | | 6.15 | |
| 2014-2018 | 11.15 | 8.00 | 7.18 | 8.90 | 6.52 | | 5.05 | | 4.88 | | 5.22 | |
|  | NHB, males | | | | | | | | | | | |
| 1989-1993 | 2.66 | 2.14 | 1.60 | 2.81 | 1.32 | | 2.17 | | 1.65 | | 2.80 | |
| 1994-1998 | 2.72 | 2.25 | 1.66 | 2.99 | 1.14 | | 1.70 | | 1.30 | | 2.19 | |
| 1999-2003 | 2.74 | 2.06 | 1.46 | 2.83 | 1.05 | | 1.44 | | 1.11 | | 1.84 | |
| 2004-2008 | 2.47 | 2.00 | 1.40 | 2.78 | 1.07 | | 1.30 | | 1.02 | | 1.64 | |
| 2009-2013 | 2.77 | 2.19 | 1.54 | 3.04 | 1.09 | | 1.19 | | 0.95 | | 1.47 | |
| 2014-2018 | 2.28 | 1.70 | 1.13 | 2.49 | 1.03 | | 1.03 | | 0.83 | | 1.26 | |
|  | NHW, females | | | | | | | | | | |  |
| 1989-1993 | 2.69 | 1.98 | 1.73 | 2.26 | 2.38 | 2.52 | | 2.38 | | 2.66 | |  |
| 1994-1998 | 2.65 | 1.94 | 1.68 | 2.23 | 2.58 | 2.68 | | 2.54 | | 2.82 | |  |
| 1999-2003 | 4.27 | 3.06 | 2.60 | 3.57 | 2.77 | 2.69 | | 2.56 | | 2.83 | |  |
| 2004-2008 | 4.19 | 3.02 | 2.53 | 3.58 | 2.85 | 2.62 | | 2.50 | | 2.76 | |  |
| 2009-2013 | 4.69 | 3.55 | 2.96 | 4.23 | 3.03 | 2.61 | | 2.48 | | 2.73 | |  |
| 2014-2018 | 4.97 | 3.71 | 3.05 | 4.48 | 2.75 | 2.18 | | 2.08 | | 2.30 | |  |
|  | NHB, females | | | | | | | | | | |  |
| 1989-1993 | 0.76 | 0.58 | 0.33 | 0.95 | 0.52 | 0.88 | | 0.59 | | 1.26 | |  |
| 1994-1998 | 0.70 | 0.58 | 0.31 | 1.00 | 0.53 | 0.79 | | 0.55 | | 1.09 | |  |
| 1999-2003 | 0.81 | 0.60 | 0.32 | 1.03 | 0.47 | 0.64 | | 0.45 | | 0.87 | |  |
| 2004-2008 | 0.75 | 0.58 | 0.29 | 1.05 | 0.44 | 0.53 | | 0.38 | | 0.73 | |  |
| 2009-2013 | 1.04 | 0.83 | 0.46 | 1.40 | 0.51 | 0.53 | | 0.39 | | 0.69 | |  |
| 2014-2018 | 0.95 | 0.72 | 0.37 | 1.29 | 0.50 | 0.48 | | 0.36 | | 0.62 | |  |

Table 4. Adjusted amenable lung and trachea cancer* mortality rates (X100,000) and 95% Confidence Intervals (CI) among NHW and NHB men by education level. United States, 1989-2018.

|  | Education level | | | | | | | | | | | |
| --- | --- | --- | --- | --- | --- | --- | --- | --- | --- | --- | --- | --- |
|  | < 12 Years | | | | ≥ 12 Years | | | | | | | |
|  | Rate | | 95%-CI | | Rate | | | | 95%-CI | | | |
| Period | Crude | Adjusted | Low | High | Crude | | Adjusted | | Low | | High | |
|  | NHW, males | | | | | | | | | | | |
| 1989-1993 | 142.04 | 92.15 | 90.64 | 93.69 | 57.24 | | 67.64 | | 66.86 | | 68.44 | |
| 1994-1998 | 151.06 | 103.46 | 101.67 | 105.27 | 58.72 | | 68.15 | | 67.40 | | 68.90 | |
| 1999-2003 | 212.75 | 128.72 | 126.23 | 131.25 | 60.28 | | 63.69 | | 62.99 | | 64.40 | |
| 2004-2008 | 209.48 | 136.49 | 133.76 | 139.26 | 58.10 | | 55.57 | | 54.96 | | 56.20 | |
| 2009-2013 | 215.28 | 140.73 | 137.75 | 143.76 | 57.28 | | 47.64 | | 47.11 | | 48.17 | |
| 2014-2018 | 206.38 | 129.50 | 126.50 | 132.58 | 52.75 | | 37.94 | | 37.51 | | 38.38 | |
|  | NHB, males | | | | | | | | | | | |
| 1989-1993 | 144.97 | 97.62 | 94.11 | 101.24 | 54.39 | | 107.33 | | 103.17 | | 111.60 | |
| 1994-1998 | 169.72 | 125.43 | 121.04 | 129.95 | 60.41 | | 113.62 | | 109.74 | | 117.60 | |
| 1999-2003 | 177.03 | 123.94 | 119.19 | 128.86 | 57.55 | | 90.78 | | 87.79 | | 93.85 | |
| 2004-2008 | 153.72 | 111.09 | 106.57 | 115.78 | 54.59 | | 77.50 | | 74.97 | | 80.09 | |
| 2009-2013 | 153.71 | 113.28 | 108.54 | 118.19 | 54.07 | | 62.74 | | 60.84 | | 64.68 | |
| 2014-2018 | 134.97 | 93.22 | 88.90 | 97.72 | 46.33 | | 46.95 | | 45.54 | | 48.40 | |
|  | NHW, females | | | | | | | | | | |  |
| 1989-1993 | 66.71 | 41.42 | 40.36 | 42.51 | 35.51 | 37.45 | | 36.92 | | 37.99 | |  |
| 1994-1998 | 80.18 | 50.04 | 48.81 | 51.29 | 40.40 | 42.17 | | 41.62 | | 42.72 | |  |
| 1999-2003 | 136.52 | 77.10 | 75.06 | 79.18 | 43.43 | 42.06 | | 41.53 | | 42.60 | |  |
| 2004-2008 | 152.32 | 87.84 | 85.52 | 90.22 | 43.89 | 42.06 | | 41.53 | | 42.60 | |  |
| 2009-2013 | 164.00 | 96.57 | 93.89 | 99.32 | 43.89 | 39.63 | | 39.13 | | 40.12 | |  |
| 2014-2018 | 166.45 | 97.00 | 94.08 | 100.00 | 42.88 | 29.80 | | 29.44 | | 30.18 | |  |
|  | NHB, females | | | | | | | | | | |  |
| 1989-1993 | 43.79 | 29.90 | 28.03 | 31.88 | 24.99 | 43.34 | | 41.12 | | 45.63 | |  |
| 1994-1998 | 59.24 | 40.38 | 38.03 | 42.86 | 30.22 | 49.98 | | 47.88 | | 52.13 | |  |
| 1999-2003 | 61.90 | 42.78 | 40.21 | 45.49 | 31.39 | 44.15 | | 42.43 | | 45.91 | |  |
| 2004-2008 | 64.15 | 45.79 | 42.99 | 48.73 | 31.60 | 40.25 | | 38.77 | | 41.78 | |  |
| 2009-2013 | 65.79 | 47.23 | 44.30 | 50.33 | 32.26 | 35.00 | | 33.79 | | 36.24 | |  |
| 2014-2018 | 62.14 | 39.84 | 37.15 | 42.70 | 29.27 | 28.24 | | 27.28 | | 29.23 | |  |

Table 5. Adjusted amenable Hodgkin’s disease* mortality rates (X100,000) and 95% Confidence intervals (CI) among NHW and NHB by gender and education level. United States, 1989-2018.

|  | Education level | | | | | | | | | | | |
| --- | --- | --- | --- | --- | --- | --- | --- | --- | --- | --- | --- | --- |
|  | < 12 Years | | | | ≥ 12 Years | | | | | | | |
|  | Rate | | 95%-CI | | Rate | | | | 95%-CI | | | |
| Period | Crude | Adjusted | Low | High | Crude | | Adjusted | | Low | | High | |
|  | NHW, males | | | | | | | | | | | |
| 1989-1993 | 0.79 | 0.66 | 0.52 | 0.83 | 0.80 | | 0.83 | | 0.75 | | 0.92 | |
| 1994-1998 | 0.67 | 0.57 | 0.43 | 0.74 | 0.67 | | 0.70 | | 0.63 | | 0.78 | |
| 1999-2003 | 1.09 | 0.91 | 0.68 | 1.20 | 0.63 | | 0.64 | | 0.57 | | 0.72 | |
| 2004-2008 | 0.95 | 0.80 | 0.57 | 1.09 | 0.55 | | 0.54 | | 0.48 | | 0.60 | |
| 2009-2013 | 1.06 | 0.88 | 0.63 | 1.22 | 0.49 | | 0.46 | | 0.41 | | 0.52 | |
| 2014-2018 | 0.86 | 0.75 | 0.49 | 1.11 | 0.43 | | 0.36 | | 0.31 | | 0.41 | |
|  | NHB, males | | | | | | | | | | | |
| 1989-1993 | 0.87 | 0.91 | 0.54 | 1.44 | 0.85 | | 1.00 | | 0.69 | | 1.41 | |
| 1994-1998 | 1.02 | 1.04 | 0.62 | 1.62 | 0.78 | | 0.92 | | 0.64 | | 1.28 | |
| 1999-2003 | 1.30 | 1.30 | 0.77 | 2.04 | 0.57 | | 0.66 | | 0.45 | | 0.93 | |
| 2004-2008 | 1.05 | 1.05 | 0.59 | 1.73 | 0.64 | | 0.72 | | 0.52 | | 0.97 | |
| 2009-2013 | 1.24 | 1.22 | 0.69 | 1.98 | 0.54 | | 0.55 | | 0.40 | | 0.74 | |
| 2014-2018 | 0.96 | 0.90 | 0.46 | 1.60 | 0.42 | | 0.42 | | 0.30 | | 0.58 | |
|  |  |  |  |  |  | |  | |  | |  | |
|  | NHW, females | | | | | | | | | | |  |
| 1989-1993 | 0.51 | 0.39 | 0.28 | 0.53 | 0.47 | 0.47 | | 0.41 | | 0.53 | |  |
| 1994-1998 | 0.50 | 0.39 | 0.28 | 0.54 | 0.46 | 0.47 | | 0.41 | | 0.53 | |  |
| 1999-2003 | 0.64 | 0.54 | 0.35 | 0.80 | 0.45 | 0.45 | | 0.39 | | 0.51 | |  |
| 2004-2008 | 0.69 | 0.63 | 0.39 | 0.94 | 0.36 | 0.45 | | 0.39 | | 0.51 | |  |
| 2009-2013 | 0.66 | 0.58 | 0.34 | 0.93 | 0.36 | 0.35 | | 0.31 | | 0.40 | |  |
| 2014-2018 | 0.74 | 0.68 | 0.40 | 1.10 | 0.24 | 0.20 | | 0.17 | | 0.24 | |  |
|  | NHB, females | | | | | | | | | | |  |
| 1989-1993 | 0.62 | 0.59 | 0.32 | 0.99 | 0.46 | 0.52 | | 0.33 | | 0.77 | |  |
| 1994-1998 | 0.60 | 0.62 | 0.31 | 1.08 | 0.45 | 0.49 | | 0.33 | | 0.71 | |  |
| 1999-2003 | 0.71 | 0.68 | 0.35 | 1.19 | 0.33 | 0.34 | | 0.22 | | 0.50 | |  |
| 2004-2008 | 0.79 | 0.75 | 0.39 | 1.31 | 0.35 | 0.37 | | 0.25 | | 0.52 | |  |
| 2009-2013 | 0.88 | 0.86 | 0.45 | 1.49 | 0.31 | 0.33 | | 0.22 | | 0.46 | |  |
| 2014-2018 | 0.64 | 0.59 | 0.25 | 1.18 | 0.24 | 0.24 | | 0.16 | | 0.35 | |  |

Table 6. Adjusted amenable leukemia* mortality rates (X100,000 and 95% Confidence intervals (CI) among NHW and NHB (25-74 years of age) by gender and education level. United States, 1989-2018.

|  | Education level | | | | | | | | | | | |
| --- | --- | --- | --- | --- | --- | --- | --- | --- | --- | --- | --- | --- |
|  | < 12 Years | | | | ≥ 12 Years | | | | | | | |
|  | Rate | | 95%-CI | | Rate | | | | 95%-CI | | | |
| Period | Crude | Adjusted | Low | High | Crude | | Adjusted | | Low | | High | |
|  | NHW, males | | | | | | | | | | | |
| 1989-1993 | 8.94 | 5.91 | 5.53 | 6.33 | 6.47 | | 7.45 | | 7.19 | | 7.72 | |
| 1994-1998 | 9.66 | 6.67 | 6.22 | 7.15 | 7.01 | | 7.96 | | 7.71 | | 8.22 | |
| 1999-2003 | 14.41 | 8.77 | 8.12 | 9.47 | 7.44 | | 7.88 | | 7.64 | | 8.14 | |
| 2004-2008 | 13.44 | 9.03 | 8.31 | 9.80 | 7.31 | | 7.16 | | 6.94 | | 7.39 | |
| 2009-2013 | 13.91 | 9.41 | 8.62 | 10.27 | 7.73 | | 6.74 | | 6.54 | | 6.95 | |
| 2014-2018 | 13.31 | 8.86 | 8.03 | 9.76 | 7.45 | | 5.68 | | 5.51 | | 5.86 | |
|  | NHB, males | | | | | | | | | | | |
| 1989-1993 | 7.24 | 5.26 | 4.43 | 6.23 | 4.82 | | 8.25 | | 7.15 | | 9.46 | |
| 1994-1998 | 9.48 | 7.25 | 6.20 | 8.44 | 5.55 | | 8.99 | | 7.95 | | 10.11 | |
| 1999-2003 | 9.28 | 6.96 | 5.81 | 8.29 | 5.49 | | 8.04 | | 7.18 | | 8.97 | |
| 2004-2008 | 10.00 | 7.48 | 6.30 | 8.84 | 5.34 | | 7.36 | | 6.60 | | 8.18 | |
| 2009-2013 | 9.14 | 7.38 | 6.12 | 8.84 | 5.82 | | 6.79 | | 6.17 | | 7.45 | |
| 2014-2018 | 8.39 | 6.48 | 5.29 | 7.89 | 5.38 | | 5.57 | | 5.09 | | 6.09 | |
|  | NHW, females | | | | | | | | | | |  |
| 1989-1993 | 5.69 | 3.60 | 3.28 | 3.94 | 4.26 | 4.44 | | 4.26 | | 4.63 | |  |
| 1994-1998 | 5.54 | 3.51 | 3.18 | 3.86 | 4.60 | 4.75 | | 4.57 | | 4.94 | |  |
| 1999-2003 | 8.33 | 4.93 | 4.39 | 5.52 | 4.75 | 4.64 | | 4.46 | | 4.82 | |  |
| 2004-2008 | 8.28 | 5.08 | 4.49 | 5.74 | 4.55 | 4.64 | | 4.46 | | 4.82 | |  |
| 2009-2013 | 8.35 | 5.26 | 4.60 | 6.00 | 4.55 | 4.23 | | 4.06 | | 4.39 | |  |
| 2014-2018 | 8.27 | 5.43 | 4.67 | 6.29 | 4.45 | 3.37 | | 3.24 | | 3.51 | |  |
|  | NHB, females | | | | | | | | | | |  |
| 1989-1993 | 4.45 | 3.19 | 2.58 | 3.91 | 3.32 | 5.05 | | 4.34 | | 5.85 | |  |
| 1994-1998 | 5.20 | 3.81 | 3.08 | 4.66 | 3.63 | 5.35 | | 4.70 | | 6.06 | |  |
| 1999-2003 | 5.22 | 3.76 | 3.00 | 4.67 | 3.81 | 4.96 | | 4.41 | | 5.56 | |  |
| 2004-2008 | 4.81 | 3.51 | 2.75 | 4.43 | 3.61 | 4.45 | | 3.97 | | 4.97 | |  |
| 2009-2013 | 5.19 | 3.91 | 3.06 | 4.93 | 3.73 | 4.06 | | 3.65 | | 4.49 | |  |
| 2014-2018 | 4.49 | 3.51 | 2.62 | 4.61 | 3.71 | 3.70 | | 3.35 | | 4.07 | |  |

Table 7. Adjusted amenable testis cancer* rates (X100,000 and 95% Confidence intervals (CI) among NHW and NHB men by education level. United States, 1989-2018.

|  | Education level | | | | | | | |
| --- | --- | --- | --- | --- | --- | --- | --- | --- |
|  | < 12 Years | | | | ≥ 12 Years | | | |
|  | Rate | | 95%-CI | | Rate | | 95%-CI | |
| Period | Crude | Adjusted | Low | High | Crude | Adjusted | Low | High |
|  | NHW | | | | | | | |
| 1989-1993 | 0.32 | 0.35 | 0.24 | 0.50 | 0.35 | 0.33 | 0.28 | 0.39 |
| 1994-1998 | 0.33 | 0.33 | 0.22 | 0.48 | 0.34 | 0.33 | 0.29 | 0.38 |
| 1999-2003 | 0.63 | 0.75 | 0.52 | 1.05 | 0.36 | 0.36 | 0.31 | 0.42 |
| 2004-2008 | 0.71 | 0.85 | 0.58 | 1.19 | 0.30 | 0.31 | 0.26 | 0.36 |
| 2009-2013 | 0.79 | 0.89 | 0.60 | 1.26 | 0.34 | 0.35 | 0.30 | 0.41 |
| 2014-2018 | 0.90 | 1.03 | 0.69 | 1.48 | 0.34 | 0.36 | 0.31 | 0.42 |
|  | NHB | | | | | | | |
| 1989-1993 | 0.15 | 0.18 | 0.04 | 0.49 | 0.14 | 0.13 | 0.05 | 0.30 |
| 1994-1998 | 0.34 | 0.37 | 0.14 | 0.78 | 0.17 | 0.14 | 0.07 | 0.29 |
| 1999-2003 | 0.19 | 0.25 | 0.05 | 0.70 | 0.22 | 0.23 | 0.12 | 0.39 |
| 2004-2008 | 0.39 | 0.45 | 0.16 | 0.97 | 0.17 | 0.16 | 0.08 | 0.28 |
| 2009-2013 | 0.12 | 0.16 | 0.01 | 0.61 | 0.16 | 0.16 | 0.08 | 0.27 |
| 2014-2018 | 0.34 | 0.38 | 0.11 | 0.92 | 0.15 | 0.16 | 0.08 | 0.26 |

Table 8. Adjusted amenable prostate cancer* mortality rates (X100,000 and 95% Confidence intervals (CI) among NHW and NHB men by education level. United States, 1989-2018

|  | Education level | | | | | | | |
| --- | --- | --- | --- | --- | --- | --- | --- | --- |
|  | < 12 Years | | | | ≥ 12 Years | | | |
|  | Rate | | 95%-CI | | Rate | | 95%-CI | |
| Period | Crude | Adjusted | Low | High | Crude | Adjusted | Low | High |
|  | NHW | | | | | | | |
| 1989-1993 | 19.44 | 10.88 | 10.42 | 11.35 | 11.96 | 14.38 | 14.01 | 14.75 |
| 1994-1998 | 18.74 | 10.75 | 10.25 | 11.27 | 10.88 | 12.98 | 12.65 | 13.32 |
| 1999-2003 | 21.36 | 10.34 | 9.74 | 10.98 | 9.20 | 10.22 | 9.93 | 10.51 |
| 2004-2008 | 18.12 | 10.30 | 9.62 | 11.02 | 8.56 | 8.62 | 8.37 | 8.87 |
| 2009-2013 | 17.39 | 10.12 | 9.39 | 10.91 | 9.06 | 7.76 | 7.54 | 7.97 |
| 2014-2018 | 17.44 | 10.04 | 9.27 | 10.88 | 10.43 | 7.43 | 7.25 | 7.63 |
|  | NHB | | | | | | | |
| 1989-1993 | 41.08 | 22.78 | 21.33 | 24.32 | 15.41 | 37.51 | 34.85 | 40.30 |
| 1994-1998 | 48.40 | 31.89 | 29.86 | 34.04 | 17.84 | 41.19 | 38.66 | 43.82 |
| 1999-2003 | 45.02 | 27.38 | 25.38 | 29.54 | 16.36 | 31.03 | 29.16 | 32.98 |
| 2004-2008 | 37.40 | 23.92 | 21.99 | 25.99 | 15.12 | 25.91 | 24.33 | 27.54 |
| 2009-2013 | 34.85 | 24.21 | 22.13 | 26.45 | 17.07 | 22.18 | 20.99 | 23.42 |
| 2014-2018 | 33.88 | 22.47 | 20.46 | 24.67 | 18.20 | 19.39 | 18.47 | 20.36 |

Table 9. Adjusted amenable breast cancer* mortality rates (X100,000) and 95% Confidence intervals (CI) among NHW and NHB by education level. United States, 1989-2018.

|  | Education level | | | | | | | |
| --- | --- | --- | --- | --- | --- | --- | --- | --- |
|  | < 12 Years | | | | ≥ 12 Years | | | |
|  | Rate | | 95%-CI | | Rate | | 95%-CI | |
| Period | Crude | Adjusted | Low | High | Crude | Adjusted | Low | High |
|  | NHW | | | | | | | |
| 1989-1993 | 33.17 | 23.09 | 22.71 | 23.48 | 31.40 | 33.75 | 33.52 | 33.98 |
| 1994-1998 | 30.96 | 21.51 | 21.12 | 21.90 | 31.10 | 32.64 | 32.42 | 32.85 |
| 1999-2003 | 43.75 | 28.81 | 28.20 | 29.43 | 29.68 | 28.60 | 28.41 | 28.80 |
| 2004-2008 | 42.61 | 28.02 | 27.38 | 28.68 | 28.29 | 25.42 | 25.24 | 25.59 |
| 2009-2013 | 41.48 | 27.73 | 27.03 | 28.45 | 28.13 | 23.32 | 23.16 | 23.48 |
| 2014-2018 | 42.29 | 28.22 | 27.43 | 29.03 | 27.32 | 21.07 | 20.92 | 21.22 |
|  | NHB | | | | | | | |
| 1989-1993 | 31.51 | 25.24 | 24.39 | 26.12 | 30.97 | 45.95 | 44.99 | 46.93 |
| 1994-1998 | 37.83 | 30.25 | 29.25 | 31.28 | 36.16 | 50.45 | 49.56 | 51.34 |
| 1999-2003 | 37.86 | 30.04 | 28.99 | 31.11 | 35.98 | 43.92 | 43.20 | 44.65 |
| 2004-2008 | 38.27 | 31.61 | 30.47 | 32.78 | 34.98 | 39.42 | 38.80 | 40.05 |
| 2009-2013 | 38.68 | 30.98 | 29.83 | 32.17 | 36.24 | 37.20 | 36.66 | 37.74 |
| 2014-2018 | 37.17 | 28.82 | 27.63 | 30.05 | 34.68 | 33.75 | 33.28 | 34.22 |

Table 10. Adjusted amenable cervix cancer* mortality rates (X100,000) and 95% Confidence intervals (CI) among NHW and NHB by education level. United States, 1989-2018.

|  | Education level | | | | | | | |
| --- | --- | --- | --- | --- | --- | --- | --- | --- |
|  | < 12 Years | | | | ≥ 12 Years | | | |
|  | Rate | | 95%-CI | | Rate | | 95%-CI | |
| Period | Crude | Adjusted | Low | High | Crude | Adjusted | Low | High |
|  | NHW | | | | | | | |
| 1989-1993 | 33.17 | 23.09 | 22.71 | 23.48 | 31.40 | 33.75 | 33.52 | 33.98 |
| 1994-1998 | 30.96 | 21.51 | 21.12 | 21.90 | 31.10 | 32.64 | 32.42 | 32.85 |
| 1999-2003 | 43.75 | 28.81 | 28.20 | 29.43 | 29.68 | 28.60 | 28.41 | 28.80 |
| 2004-2008 | 42.61 | 28.02 | 27.38 | 28.68 | 28.29 | 25.42 | 25.24 | 25.59 |
| 2009-2013 | 41.48 | 27.73 | 27.03 | 28.45 | 28.13 | 23.32 | 23.16 | 23.48 |
| 2014-2018 | 42.29 | 28.22 | 27.43 | 29.03 | 27.32 | 21.07 | 20.92 | 21.22 |
|  | NHB | | | | | | | |
| 1989-1993 | 31.51 | 25.24 | 24.39 | 26.12 | 30.97 | 45.95 | 44.99 | 46.93 |
| 1994-1998 | 37.83 | 30.25 | 29.25 | 31.28 | 36.16 | 50.45 | 49.56 | 51.34 |
| 1999-2003 | 37.86 | 30.04 | 28.99 | 31.11 | 35.98 | 43.92 | 43.20 | 44.65 |
| 2004-2008 | 38.27 | 31.61 | 30.47 | 32.78 | 34.98 | 39.42 | 38.80 | 40.05 |
| 2009-2013 | 38.68 | 30.98 | 29.83 | 32.17 | 36.24 | 37.20 | 36.66 | 37.74 |
| 2014-2018 | 37.17 | 28.82 | 27.63 | 30.05 | 34.68 | 33.75 | 33.28 | 34.22 |

Table 11. Adjusted amenable body of uterus cancer* mortality rates (X100,000) and 95% Confidence intervals (CI) among NHW and NHB female by education level. United States, 1989-2018.

|  | Education level | | | | | | | |
| --- | --- | --- | --- | --- | --- | --- | --- | --- |
|  | < 12 Years | | | | ≥12 Years | | | |
|  | Rate | | 95%-CI | | Rate | | 95%-CI | |
| Period | Crude | Adjusted | Low | High | Crude | Adjusted | Low | High |
|  | NHW | | | | | | | |
| 1989-1993 | 4.74 | 2.68 | 2.43 | 2.96 | 3.14 | 3.28 | 3.12 | 3.44 |
| 1994-1998 | 4.67 | 2.85 | 2.56 | 3.16 | 3.41 | 3.55 | 3.39 | 3.71 |
| 1999-2003 | 6.54 | 3.68 | 3.25 | 4.17 | 3.96 | 3.82 | 3.66 | 3.99 |
| 2004-2008 | 7.70 | 4.53 | 4.00 | 5.12 | 4.36 | 3.90 | 3.74 | 4.05 |
| 2009-2013 | 8.36 | 5.15 | 4.52 | 5.86 | 5.44 | 4.33 | 4.18 | 4.48 |
| 2014-2018 | 9.94 | 6.14 | 5.38 | 6.99 | 6.65 | 4.69 | 4.54 | 4.84 |
|  | NHB | | | | | | | |
| 1989-1993 | 7.63 | 4.67 | 4.00 | 5.45 | 3.73 | 6.93 | 6.03 | 7.92 |
| 1994-1998 | 8.67 | 5.45 | 4.64 | 6.37 | 4.51 | 7.72 | 6.90 | 8.60 |
| 1999-2003 | 9.65 | 6.04 | 5.15 | 7.06 | 5.26 | 7.66 | 6.95 | 8.42 |
| 2004-2008 | 9.94 | 6.59 | 5.60 | 7.72 | 6.30 | 8.30 | 7.62 | 9.01 |
| 2009-2013 | 11.82 | 8.10 | 6.94 | 9.43 | 8.42 | 9.17 | 8.55 | 9.81 |
| 2014-2018 | 13.94 | 8.77 | 7.52 | 10.20 | 10.84 | 10.34 | 9.76 | 10.94 |

Table 12. Adjusted amenable other type cancers* mortality rates (X100,000) and 95% Confidence intervals (CI) among NHW and NHB by gender and education level. United States, 1989-2018.

|  | Education level | | | | | | | | | | | |
| --- | --- | --- | --- | --- | --- | --- | --- | --- | --- | --- | --- | --- |
|  | < 12 Years | | | | ≥ 12 Years | | | | | | | |
|  | Rate | | 95%-CI | | Rate | | | | 95%-CI | | | |
| Period | Crude | Adjusted | Low | High | Crude | | Adjusted | | Low | | High | |
|  | NHW, males | | | | | | | | | | | |
| 1989-1993 | 106.71 | 71.19 | 69.82 | 72.58 | 66.17 | | 77.63 | | 76.79 | | 78.48 | |
| 1994-1998 | 117.17 | 83.58 | 81.92 | 85.26 | 73.50 | | 83.94 | | 83.11 | | 84.77 | |
| 1999-2003 | 175.77 | 113.70 | 111.26 | 116.19 | 82.13 | | 85.39 | | 84.58 | | 86.21 | |
| 2004-2008 | 179.45 | 124.66 | 121.95 | 127.43 | 84.89 | | 80.15 | | 79.42 | | 80.90 | |
| 2009-2013 | 200.10 | 138.75 | 135.68 | 141.89 | 94.40 | | 78.92 | | 78.24 | | 79.60 | |
| 2014-2018 | 225.49 | 149.53 | 146.14 | 152.98 | 102.36 | | 76.00 | | 75.38 | | 76.64 | |
|  | NHB, males | | | | | | | | | | | |
| 1989-1993 | 144.66 | 104.34 | 100.53 | 108.27 | 65.87 | | 121.78 | | 117.42 | | 126.26 | |
| 1994-1998 | 176.30 | 136.97 | 132.24 | 141.84 | 78.43 | | 135.77 | | 131.63 | | 140.00 | |
| 1999-2003 | 187.25 | 139.20 | 133.96 | 144.60 | 80.87 | | 119.26 | | 115.91 | | 122.68 | |
| 2004-2008 | 178.56 | 136.59 | 131.38 | 141.96 | 82.74 | | 109.86 | | 106.93 | | 112.84 | |
| 2009-2013 | 192.02 | 148.01 | 142.38 | 153.81 | 91.42 | | 102.06 | | 99.68 | | 104.47 | |
| 2014-2018 | 196.76 | 142.27 | 136.70 | 148.04 | 92.20 | | 91.93 | | 89.98 | | 93.92 | |
|  | NHW, females | | | | | | | | | | |  |
| 1989-1993 | 75.09 | 45.21 | 44.11 | 46.34 | 50.11 | 52.79 | | 52.16 | | 53.43 | |  |
| 1994-1998 | 77.50 | 47.99 | 46.79 | 49.23 | 55.21 | 57.47 | | 56.84 | | 58.12 | |  |
| 1999-2003 | 119.06 | 68.62 | 66.64 | 70.65 | 58.78 | 56.96 | | 56.34 | | 57.58 | |  |
| 2004-2008 | 120.39 | 71.85 | 69.67 | 74.09 | 57.94 | 52.52 | | 51.95 | | 53.09 | |  |
| 2009-2013 | 125.21 | 77.04 | 74.55 | 79.60 | 61.65 | 50.12 | | 49.60 | | 50.65 | |  |
| 2014-2018 | 135.40 | 83.91 | 81.03 | 86.87 | 65.81 | 47.67 | | 47.19 | | 48.16 | |  |
|  | NHB, females | | | | | | | | | | |  |
| 1989-1993 | 75.18 | 51.00 | 48.54 | 53.56 | 44.16 | 74.80 | | 71.88 | | 77.80 | |  |
| 1994-1998 | 91.23 | 63.45 | 60.44 | 66.58 | 51.69 | 82.42 | | 79.75 | | 85.16 | |  |
| 1999-2003 | 88.95 | 61.87 | 58.75 | 65.14 | 53.35 | 73.11 | | 70.91 | | 75.35 | |  |
| 2004-2008 | 86.01 | 62.09 | 58.79 | 65.53 | 52.58 | 65.48 | | 63.60 | | 67.40 | |  |
| 2009-2013 | 90.75 | 66.43 | 62.87 | 70.15 | 57.76 | 62.09 | | 60.49 | | 63.73 | |  |
| 2014-2018 | 94.79 | 64.70 | 61.06 | 68.52 | 61.38 | 59.33 | | 57.93 | | 60.74 | |  |
